# Effectiveness of dietetic care for cancer survivors in the primary care setting: A systematic review and meta-analysis of randomized controlled trials

**DOI:** 10.1007/s11764-024-01583-6

**Published:** 2024-05-06

**Authors:** Henriette G. Ryding, Lana J. Mitchell, Roshan R. Rigby, Lauren Ball, Julie Hobby, Lauren T. Williams

**Affiliations:** 1 Griffith University, Brisbane, Australia; 2 Menzies Health Institute Queensland, Southport, QLD Australia; 3 University of Queensland, Brisbane, Australia

**Keywords:** Dietitian, Dietetic consultation, Nutritional care, Cancer, Cancer survivorship, Primary health care

## Abstract

**Purpose:**

Nutrition plays an important role in cancer survivorship. This systematic review and meta-analysis aim to critically assess and quantify the effectiveness of nutrition care interventions provided by dietitians to survivors who have completed treatment for cancer.

**Methods:**

A systematic review of randomized controlled trials (RCTs) published from January 2004 to November 2023 reporting the effectiveness of primary care dietetic interventions with adult cancer survivors was conducted. PubMed, Scopus, CINAHL, Embase, ProQuest and PsycINFO databases were searched for key terms. Meta-analyses were conducted where there were sufficient studies of the same cancer type and outcomes.

**Results:**

Twelve RCTs representing 1138 cancer survivors (519 breast cancer; 75 prostate cancer; 544 colorectal cancer) were included. Primary outcome measures included weight loss *(n* = *6),* quality of life *(n* = *2),* reducing lymphedema-related arm volume *(n* = *2),* nutritional status *(n* = *1)* and increasing fruit and vegetable intake *(n* = *1)*. Weight loss was observed in studies where this was the primary outcome. Results for quality of life varied. Meta-analyses of RCTs with breast cancer survivors showed that dietitian intervention achieved a mean of 3.7 kg greater intentional weight loss and 2.3% greater body fat decrease than control *(p < 0.0001).*

**Conclusions:**

This study provides evidence for the effectiveness of primary care dietetic interventions by dietitians with cancer survivors, particularly with respect to intentional weight and fat loss in breast cancer survivors.

**Implications for cancer survivors:**

Dietitians can play a key role in managing weight and improving long term health outcomes and prognosis for cancer survivors beyond the acute care setting.

**Supplementary Information:**

The online version contains supplementary material available at 10.1007/s11764-024-01583-6.

## Background

The incidence of cancer is rapidly increasing worldwide [1] and the number of people living with and beyond a cancer diagnosis has risen proportionately [2]. A cancer survivor is defined as someone who has received a cancer diagnosis, starting at the time of diagnosis and lasting through until end of life [3]. In 2018, there were an estimated 43.8 million cancer survivors worldwide [4]. Of these, 18.1 million cancer survivors live in the United States [5], three million in the United Kingdom [6] and more than one million in Australia [7], with these numbers expected to increase significantly over the next two decades [5–7]. Those who survive initial treatment for cancer have ongoing health challenges including late effects [8] of the cancer itself and/or the cancer treatment therapies [9]. Cancer survivors are also more likely to have had or to develop chronic disease when compared to the population without cancer [10] and to have an increased risk of cancer recurrence or development of new cancers [11]. These health issues can be managed or ameliorated by modification of lifestyle factors such as dietary intake [12].

Cancer agencies target their lifestyle and nutrition guidelines towards cancer prevention or cancer survivorship. The American Cancer Society Guidelines for Cancer Survivors focuses on general and cancer-site-specific advice for cancer survivors, including the recommendation that survivors achieve and maintain a healthy body weight [13]. Cancer survivors presenting with involuntary weight loss [14] or excess weight gain generally have a worse prognosis [15], warranting additional support through nutritional care [16]. The prevention-oriented World Cancer Research Fund (WCRF) [17] guidelines are also relevant to cancer survivors given their increased risk of developing new primary cancers [11]. The WCRF guidelines recommend a diet rich in whole grains, vegetables, fruit, and beans, and low in red meat, processed meat and sugar-sweetened beverages while maintaining a healthy weight [18]. Cancer survivors can be supported in making these dietary changes through provision of nutritional care.

Dietitians are health professionals qualified to apply evidence-based, nutritional care to promote and optimize health while preventing and treating diseases [19]. A systematic review of randomized controlled trials (RCTs) conducted by Mitchell and colleagues investigated the effectiveness of dietetic consultations delivered in the primary health care setting to people with chronic disease [20]. Two of the included studies focused on intervention with cancer patients in the active treatment phase. One study showed significant improvement in nutrition-related symptoms in colorectal cancer patients undergoing radiotherapy [21]. The other study found that dietitians helped breast cancer patients to decrease their energy intake to prevent weight gain associated with chemotherapy [22].

The importance of dietetic care during cancer treatment, delivered as part of a multidisciplinary team based in an acute care setting, is well documented [23]. However, given the increasing number of long-term cancer survivors [24], investigation into how to support survivors beyond the acute care service phase is warranted. Systematic literature reviews have been conducted regarding the diet and weight management of cancer survivors [25–28], however, none of these reviews have focused specifically on care provided by primary care dietitians. This systematic review and meta-analysis aim to synthesize RCTs critically assessing and quantifying the effectiveness of nutritional care interventions provided to cancer survivors by dietitians in primary care.

## Methods

A systematic review and meta-analysis of RCTs was chosen to provide a comprehensive and clear outline of the highest level of evidence available and to aid in recognizing gaps in this field of research [29]. This systematic review and meta-analysis of RCTs adhered to the Preferred Reporting Items for Systematic Reviews and Meta-Analyses (PRISMA) guidelines [30]. The prospective protocol was registered with the International Prospective Register of Systematic Reviews (PROSPERO; CRD42023437064).

### Search strategy

A comprehensive search of peer-reviewed literature was conducted between March and April 2023 for studies published between January 2004 and February 2023 and updated in November of 2023 using PubMed, Scopus, CINAHL (via EBSCOhost), Embase, ProQuest and PsycINFO. The date of January 2004 was chosen to capture data published after the seminal reports on cancer survivorship: ‘National Action Plan for Cancer Survivorship’ [31], ‘Living Beyond Cancer: Finding a New Balance’ [32], and from the Institute of Medicine ‘From Cancer Patient to Cancer Survivor: Lost in Transition’ [3]. This last work identified essential components of cancer survivorship plans post active treatment including care co-ordination, prevention and surveillance of new and recurrent cancers, and intervention for long term and late effects of cancer.

A structured search strategy was developed in consultation with an experienced university research librarian. Boolean operators OR and AND were used and MeSH terms and non-MeSH terms were applied to narrow the focus. Titles and abstracts with at least one search term deriving from the following three categories were included for screening: ‘neoplasm OR cancer OR oncology OR survivor* OR cancer survivor* OR oncology survivor’ AND ‘dietitian OR dietician OR dietetics OR nutritionist OR diet advice OR nutrition advice’ AND ‘consult* OR referral OR private practice OR counsel* OR interview OR advice OR outpatient OR clinic OR primary care OR primary health care OR community health’. Studies were limited to interventions with adults ≥ 18 years of age and to the English language. The reference lists of systematic reviews were hand-searched for papers not captured by the preliminary search strategy.

### Study selection

All citations retrieved during the search process were exported into Covidence [33], a web-based systematic review screening and extraction platform, and duplicates were removed. Studies were screened using the Population-Intervention-Comparator-Outcome-Study (PICOS) framework for the inclusion criteria.

- Population: Adult (≥ 18 years) survivors of all cancer types who have completed the active treatment phase of cancer treatment (surgery, radiology, chemotherapy).
- Intervention: Nutritional care provided exclusively by a dietitian in a primary care setting including community, and private practice.
- Comparator: Usual care, minimal care, or no intervention control.
- Outcome: Anthropometric measures: weight, height, Body Mass Index (BMI), waist circumference, waist-to-hip ratio, skinfold thickness. Clinical indicators: biomarkers: cholesterol, triglycerides, blood glucose levels, inflammation, carotenoids. Dietary intake, dietary behaviors. Quality of Life (QoL).
- Study: RCTs

A pilot screening of the title and abstract for 100 studies was conducted independently by four researchers who then met to moderate, discuss any inconsistencies and further elucidate the PICOS criteria. Citations were screened in duplicate by title and abstract. Full-text versions of studies that appeared to meet the inclusion criteria were retrieved and screened in duplicate to determine their eligibility for inclusion. Conflicts were discussed among the research team until agreement was achieved.

### Data extraction

Information extracted from eligible studies included author/s, year published, country, study aim, setting, study design, assessment tools, role of dietitian, participant characteristics (age, eligibility), type of cancer, dietary goals of intervention, duration of intervention and intensity, control arm description and comparators. Study outcome measures relevant to cancer survivors (anthropometric, clinical, dietary intake and quality of life) were recorded. Interventions were described as effective if a statistically significant (*p* ≤ 0.05) difference in the primary outcome measure between intervention and control was observed at the end of the intervention. The threshold for achieving clinical significance in weight loss was set at a loss of 5% from baseline as per the nutrition and physical activity guidelines for cancer survivors that note achieving at least 5% as having significant health benefits[13]. Data were extracted only from study arms that met the inclusion criteria. Data extraction was conducted by HR and checked against the original articles by LTW. The Select Statistical Services online tool for two sample t-test was used to calculate *p* value in studies that did not provide these data. If the* p*-values could not be calculated from the data provided, they were requested from the original study authors.

### Quality assessment

Quality assessment was conducted using the Cochrane tool 1 for risk of bias in randomized trials [34], in conjunction with Covidence [33]. This tool does not allocate an overall risk assessment to individual studies [34]. The following six domains are each rated as high, low, or unclear: (1) sequence generation, (2) allocation concealment, (3) blinding of participants and personnel, (4) blinding of outcome assessors, (5) incomplete outcome (6) selective outcome reporting. Two reviewers (HR, JH) independently assessed each included study for quality. Inconsistencies were discussed with a third researcher (RR) until consensus was reached.

### Meta-analysis

Meta-analysis was performed if there were at least two studies that reported sufficiently homogenous outcome measures and cancer type [35]. Meta-analysis was conducted using Jamovi software version 2.3 [36]. The analysis was carried out using mean difference as the outcome measure. A random-effects model was chosen due to the variability of intervention design [35]. The amount of heterogeneity (i.e., tau^2^), was estimated using the restricted maximum-likelihood estimator [37]. In addition to the estimate of tau^2^, the Q-test for heterogeneity (Cochran 1954) and the I^2^ statistic are reported. The I^2^ test is a heterogeneity test, expressed as a percentage [38], where an I^2^ value of 0% indicates no heterogeneity and 100% indicates maximum heterogeneity [38]. If heterogeneity is detected (i.e., tau^2^ > 0, regardless of the results of the Q-test), a prediction interval for the true outcomes is also provided [39]. Studentized residuals and Cook's distances are used to examine whether studies may be outliers and/or influential in the context of the model [40]. Studies with a studentized residual larger than the 100 x (1—0.05/(2 X k))th percentile of a standard normal distribution are considered potential outliers (using a Bonferroni correction with two-sided alpha = 0.05 for k studies included in the meta-analysis) [41]. Studies with a Cook's distance larger than the median plus six times the interquartile range of the Cook's distances are considered to be influential [40]. The rank correlation test and the regression test, using the standard error of the observed outcomes as predictor, are used to check for funnel plot asymmetry [42]. When standard deviation was not reported within the study results, it was calculated from the 95% confidence interval and in one study from the standard error [43].

## Results

**Fig. 1:**
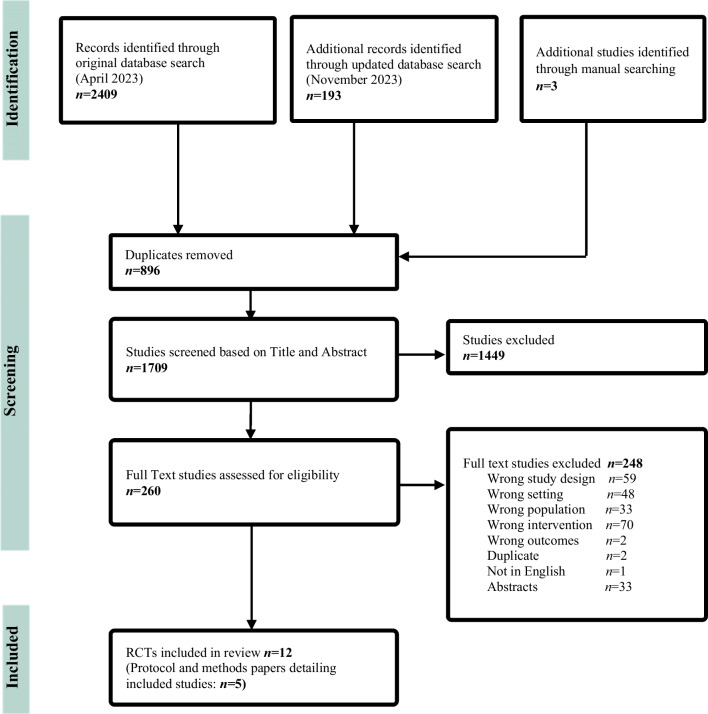
Flow diagram for systematic review depicting data screening

The database search identified 2602 records and hand searching identified a further three records. The PRISMA [44] flow chart depicting the study selection process is shown in Fig. 1. Following duplicate removal, title and abstract screening and full text screening, 17 papers representing 12 unique RCTs were eligible for inclusion. One study [45] had two intervention endpoints and data from both were included. The characteristics of the included RCTs grouped by cancer site are described in Table 1. Details of the interventions and outcome measures of the RCTs are summarized in Table 2.

**Table 1:** Description of included randomized controlled trials

Author (year) Ref#	Country; Setting	Study aim/s	Sample characteristics	Outcome Measures (measurement tools) Primary outcome measure ^a^				Sample (n)		
				Anthropometry	Biochemistry	Dietary intake	Clinical and Quality of Life	Baseline total (n) I:C (n)	Analyzed total (n) I:C (n)	Dropout total (%) I:C (%)
Breast Cancer										
Cho et al. (2014) [46]	Korea Outpatients	To implement an intensive nutrition intervention, to increase fruit and vegetable intake and evaluate changes in serum antioxidant levels and QoL	- Women - 25–65 mean (SD) 46.1 (7.5) y - Completed Stage I-III treatment - Healthy weight: BMI < 25 kg/m2 - Without: uncontrolled disease or BMI ≥ 25 kg/m2 - Recruited from previous breast cancer patients of Seoul Hospital	- Weight (scales) - Height (stadiometer) - WC	- Serum antioxidants (HPLC)	- F&V intake ^a^ (3-day food diary analyzed with CAN-pro-3.0, Korea)	- Functional Assessment of Cancer Therapy (FACT-B) [47]	86 48:38	61 30:31	29 18:37.5
Harrigan et al. (2016) [48]	United States of America Yale Cancer Centre Survivorship Clinic	To compare 6-month weight loss counselling (face to face or telephone) with usual care on weight with secondary measures of body composition, physical activity, diet & serum biomarkers	- Women - mean (SD) 59 (7.5) y - 3 m post active treatment for Stage 0-III diagnosed within 5y - Overweight: BMI ≥ 25.0 kg/m^2^; mean (SD) 33.1 (6.6) kg/m^2^ - Able to exercise - Without: pregnancy, recent stroke or MI, mental illness - Recruited from 5 Connecticut hospitals through Connecticut Tumor Registry or self-referred through Yale-New Haven and Yale Cancer Centre Survivorship clinic	- Weight ^a^ - Height (stadiometer) - BMI - HC - WC - Body fat (DEXA -Hologic 4500 scanner)	- Serum biomarkers (Radioimmunoassay kits; Automated chemistry analyzer)	- Changes in EI - % EI as Fat - Fiber - F&V - Added sugar (120 item Women’s Health Initiative FFQ; University of Minnesota Nutrition Coding Centre Nutrient Database)	NM	100 Int 1: 33 Int 2: 34 C: 33	85^b^ Int 1: 30 Int 2: 24 C: 31	15 9 29 6
Jen et al. (2004) [49] Djuric et al (2002) [50]	United States of America Community	To compare efficiency of weight loss regimens on weight and metabolic improvement in BC survivors (4 arms –only Individualized arm and control reported here)	- Women - 18–70 y; mean (SD) 51.7 (8.4) y - ≥ 3 m post active treatment (exception: hormone therapy) - Stage I < 2 cm tumor or stage II ˃2 cm tumor diagnosed < 4 y - Obese: BMI ˃ 30 kg/m^2^; mean (SD) 35.2 (1.2) kg/m^2^ - Recruited through Race for the Cure mailout, press releases or breast clinic brochures	- Weight (professional beam scale) - BMI - % body fat (tetrapolar bioelectrical impedance)	- Serum biomarkers ^a^ (Enzyme immunoassays kits; Radioimmunoassay kits; Spectrophotometric assay with glucose oxidase)	- EI - % E as fat (3-day food record analyzed with Minnesota Nutrition Data; System Research Software using Food database version 14A)	NM	26 13:13	21 9:12	19 31:8
Reeves et al. (2017) [51]	Australia Community	To assess the feasibility, efficacy, acceptability, and safety of a telephone delivered weight loss intervention for BC survivors	- Women - 18–75 mean (SD) 55.3 (8.7) y - Post active treatment for - Stage I-III diagnosed 9–18 m prior - Overweight/Obese: BMI 25–40 kg/m^2^; mean (SD) 31.0 (4.3) kg/m^2^ - Without: ductal carcinoma, invasive BC, other cancer in last 5 y - Recruited from state-based cancer registry in Queensland	- Weight change (electronic scale) - Height (stadiometer) - HC - WC (non- expandable tape measure) - Fat mass and Fat Free Mass (bioimpedance spectroscopy)	NM	NM	- QoL (SF-36 physical and mental component scores) [52] - Fatigue (FACIT-F)[53] - Body Image (BIRS)[54]	90 45:45	74^b^ 40:34	18 11:15
Reeves et al. (2021) [55] Reeves et al (2016) [56]	Australia Community	To evaluate the effectiveness of a remotely delivered weight loss intervention in BC survivors	- Women - 18–75 mean (SD) 55.4 (9.2) y - Post active treatment (exception: endocrine treatment) - Stage I-III diagnosed within 2 y - Overweight/Obese: BMI 25–45 kg/m^2^; mean (SD) 31.4 (5.1) kg/m^2^ - Without: pregnancy, contra-indications to unsupervised exercise, ˃5% weight loss in last 6 m - Recruited from 7 Brisbane hospitals and state-based registry in Queensland	- % Weight change ^a^ (Tanita calibrated scales) - Height (stadiometer) - HC - WC (non- expandable tape measure) - Body composition (DEXA Lunar Prodigy)	- Serum biomarkers (Triglyceride; HDL-C; Fasting Plasma Glucose; Metabolic syndrome risk score)	NM	- Hand grip (handheld dynamometer) - BP (automated sphygmomanometer) - Menopause symptoms (Greene Climacteric Scale)[57] - Arthralgia (Musculoskeletal Pain (subscale from Breast Cancer Prevention Trial Symptom Scale)[58] - QoL (PROMIS[59], FACIT-F)[53] - Body image (BIRS) [54] - Fear of Cancer recurrence (CARQ-4)[60]	159 79:80	130^b^ 70:60	18 11:25
Shaw et al. (2007a) [61]	United Kingdom Community	To evaluate effect of 2 dietary interventions (weight reduction and low-fat) on BC related lymphedema	- Women - Age: no criterion; Median age 65 y - 12 m post active treatment - ± hormone treatment - BMI: No criterion; group mean NR but means for each group > 25 kg/m^2^ - Remission from cancer - Swollen arm ≥ 20% excess volume compared to unaffected arm - Recruited through Lymphedema Clinic, Royal Marsden Hospital	- Weight (Seca digital scales) - Height - Skinfold thickness -unaffected arm (Harpenden calipers)	NM	- EI - Nutrient intake (7-day dietary diaries with photos analyzed using Dietplan 5 software based on McCance & Widdowson’s Composition of Foods, UK)	- Excess arm volume ^a^ % affected arm volume compared with non-affected arm volume (Pedometer; arm circumference by tape measure to calculate volume)	64^d^ Int 1:NR Int 2:NR C: NR	51^b^ Int 1: 19 Int 2: 17 C: 15	20 Int 1:NR Int 2:NR C:NR
Shaw et al. (2007b) [62]	United Kingdom Community	To evaluate the effectiveness of weight change in women with BC related lymphedema relative to excess arm volume	- Women - 18–75 y; mean (SD) - 12 m post active treatment - ± hormone treatment - BMI ≥ 25 kg/m^2^; group mean NR but means for each group > 25 kg/m^2^ - Remission from cancer - Received compression treatment for arm lymphedema from BC treatment - Swollen arm ≥ 15% excess volume of unaffected arm - Recruited through Lymphedema Clinic, Royal Marsden Hospital; Lymphedema Support Network	- Weight (digital scales) - Height (stadiometer) - Skinfold thickness -unaffected arm (Harpenden calipers)	NM	- EI - protein - fat - carbohydrate (7-day dietary diaries with photos analyzed using Dietplan5 software based on McCance & Widdowson composition of Foods, UK)	- Excess arm volume ^a^ % affected arm volume compared with non-affected arm volume (arm circumference by tape measure to calculate volume)	24^d^ NR	21^b^ 11:10	12.5 NR
**Prostate Cancer**										
Baguley et al. (2020) [63] Baguley et al (2017) [64]	Australia Community	To evaluate the effects of a 12-week MED-diet, on cancer related fatigue (CRF) and QoL in men with PC	- Men - ≥ 18 y; mean (SD) 65.9 (7.8) y - ≥ 3 m treatment with Androgen Deprivation Therapy - BMI 18.5–34.9 kg/m^2^; mean (SD) 28.9 (3.4) kg/m^2^ - Non-smoker - Without: current infection, bone metastases or any condition preventing safe completion of study - Recruited through Mater Adult Hospital, University of Queensland and Cancer Council, Queensland	- BMI - Height - Body composition (DEXA: Hologic)	- Fasting Inflammatory markers: IL-6 and IL-8 (ELISA kit)	- Changes in Dietary Intake (1 month diet history using Wollongong Dietary Inventory)	- CRF^a^ (FACIT-F) [53] - QoL^a^ (FACIT-G [53]; - SF-36 [52])	23 12:11	21 11:10	8.7 8:9
Mohamad et al. (2019) [65]	United Kingdom Home-based	To test effect of 12- week diet and physical activity intervention on body weight and QoL in overweight and obese men treated for PC	- Men - 48–81 y; mean (SD) 65.5 (5.6) y - Localized or locally advanced PC diagnosed within 3 y - BMI ≥ 25 kg/m^2^ in men < 70 y - BMI ≥ 30 kg/m^2^ in men ≥ 70 y - Without: distant metastases, other weight management program - Recruited through the Urological Cancer database	- Change in weight ^a^ (SECA digital scales)	NM	NM	- QoL (EORTC QLQC30 [66]; - EORTC QLQ-PR25 [67])	54 26:28	54^b^ 26:28	0 0:0
Colorectal Cancer										
Alavi et al. (2023) [45] Henriksen et al. (2017) [68]	Norway Home and study center	To investigate the effect of a 6- and 12-month intensive dietary intervention on weight and body composition of CRC survivors	- Men and women - 50–80 y; mean (SD) 66.5 (7.5) y - 2–9 m post-surgery - Stage I-III recently diagnosed primary adenocarcinoma - BMI: no criterion; mean (SD) 27 (4.6) kg/m^2^ - Without: colorectal adenoma, carcinoid, abdominal, carcinomatosis or sarcoma - Participants in 2012–20 CRC-NORDIET study recruited through Oslo University Hospital and Akershus University Hospital	- Weight ^a^ (digital scale) - Height (stadiometer) - Body composition^a^: FFM, FM, FM%, VAT, SAT (Lunar iDXA) - Ratio of FM/FFM^a^	NM	NM	- PG-SGA (Norwegian version)	383 192:191	383^c^ 192:191	0 0:0
Ho et al. (2020) [69] Ho et al. (2013) [70]	China Regional centers in Hong Kong	To assess the effects of dietary and PA interventions on QoL, anxiety, and depression levels among adult Chinese CRC survivors (4 arms – only 2 arms reported here: Dietary only and control)	- Men and women - ≥ 18 y; mean (SD) 65.4 (9.6) y - Up to 1 y post active treatment - Confirmed adenocarcinoma - BMI: no criterion; mean (SD) 23.95 (3.4) kg/m2 - Without: recurrent disease, hereditary CRC syndromes, wheelchair bound, chronic HF - Recruited through 4 public hospitals in Hong Kong	- BMI - Weight (calibrated scale) - Height (stadiometer) - HC (tape measure) - WC (Tape Measure) - Body & visceral fat (Bioelectrical Impedance)	- Anemia (Hemoglobin)	- Red/processed meat - Refined grain (Shanghai Women’s Health Study FFQ) [71]	- General QoL ^a^ (SF-6D [72], SF-12 [73, 74], - CRC related QoL^a^ (FACT-C [75] FACT-G) [76], - Anxiety and depression^a^ (HADS) [77]	112 56:56	101 48:53	10 14:5
Wang et al. (2022) [78]	China Changning District Beixinjing Community Health Service Centre	To evaluate the effects of personalized nutrition intervention on nutritional status and QoL of CRC survivors	- Men and women - ≥ 18 y; mean (SD) 68.3 (7.9) y - CRC Diag Sep 1, 2015-July 31, 2020 - Able to communicate and eat independently - Life expectancy ˃6 m - PG-SGA ≥ 2 - BMI: no criterion; mean (SD) 23.8 (3.1) kg/m^2^ - Without severe heart, lung or kidney disease, metastatic diseases and recurrent tumors, poor compliance - Recruitment: Colorectal cancer survivors living in Beixinjing Community, Changning Province	- BMI - Weight - Height - Calf circumference	NM	- Dietary intake: E, protein, fat, intake (24-h diet recall questionnaire analyzed with - Dietary survey software, Shanghai Zhending)	- Malnutrition status ^a^ (PG-SGA) - QoL (EORTC QLQ-C30) [66]	60 30:30	56^b^ 28:28	7 7:7

^a^Primary outcome measure

^b^Intention to treat analysis conducted

^c^All participants were analyzed for weight. Number of participants analyzed for body composition: Total: 319; I:C 157:162

^d^Did not provide breakdown of participant numbers into intervention and control groups at baseline

Abbreviations: BC = Breast cancer; BIRS = Body image and relationships scale; BMI = Body mass index; BP = Blood pressure; C = Control; CARQ-4 = Concerns about recurrence questionnaire; CRC = Colorectal cancer; CRF = Cancer related fatigue; DEXA = Dual-energy x-ray absorptiometry scans; E = Energy; EI = Energy intake; EORTCQLQ-C30 = European organization for research and treatment of cancer for assessing the generic aspects of QoL; EORTCQLQ-PR25 = European organization for research and treatment of cancer specific for prostate cancer; FACIT-F = Functional assessment of chronic illness therapy—fatigue; FACIT-G = Functional assessment of chronic illness therapy – general; FACT-B: Functional assessment of cancer therapy-breast cancer; FACT-C = Functional assessment of cancer therapy-colorectal scale; FACT-G = Functional assessment of cancer therapy–general; FFQ = Food frequency questionnaire; FM = Fat mass; FFM = Fat free mass; F = Fruit; HADS = Hospital anxiety and depression scale; HC: Hip circumference; HDL-C = High density lipoprotein cholesterol; HF = Heart failure; HPLC = High-performance liquid chromatography; I = Intervention; IL-6 = Interleukin-6; IL-8 = Interleuken-8; Int = Intervention; MED-diet = Mediterranean-style eating pattern; MI = Myocardial infarction; m = month; NM = Not measured; NR = Not reported; PC = Prostate cancer; PG-SGA = Patient generated – subjective global assessment; PROMIS = Patient-Reported Outcome Measurement Information System; QoL = Quality of life; SAT = Subcutaneous adipose tissue; SD = Standard deviation; SF-12 = 12-item short form health survey; SF-36 = 36-item short-form health survey; SF-6D = Six-dimensional health state short form; V = Vegetable; VAT = Visceral adipose tissue; WC = Waist circumference; y = year

**Table 2:** Description of interventions, outcome measures and effectiveness of included randomized controlled trials

Study	Intervention				Control	Study outcome measure comparison between intervention and control at data end point (primary outcome^a^)				Effectiveness for primary outcome Y/N
Author (year)	Intervention duration; Study data end point	Behavior change framework; Dietitian strategy; Dietitian time	Dietary goals	Reference to nutrition guidelines		Anthropometry	Biochemistry	Dietary intake	Clinical and QoL	
Breast Cancer										
Cho et al. (2014) [46]	8 weeks; 8 weeks	NR Individual nutritional counselling Cooking class 1 × 60 min 2 × 40 min over 8 weeks = 140 min	- Eat at least 10 serves of F&V/day	NR	Print material (brochure and general dietary guideline)	- Body weight (kg): NS - BMI (kg/m^2^): NS - WC (cm): NS - Weight loss (%) NCS	Serum antioxidant levels - Carotene (µg/dL) *** - Vitamin A (mg/L) * - Vitamin E (mg/L**): NS**	Fruit ^a,b^ (g/d) I: Wk0 266.0 I: Wk8 331.5 p < 0.001 Vegetable ^a,b^ (g/day) I: Wk0 410 I: Wk8 716 p < 0.001 C: NS	QoL: - FACT-B: NS	Unable to calculate from data provided
Harrigan et al. (2016) [48]	6 months; 6 months	Theory of Planned Behavior [79]; Individualized counselling Education in portion size and mindful eating practices **Int 1**: In person 11 × 30 min over 6 months = 5.5 h **Int 2**: Telephone 11 × 30 min over 6 months = 5.5 h	Weight loss by: “LEAN” program - E deficit of ~ 500 kcal/day - EI: 1200–2000 kcal/day - Fat < 25% of E - Predominantly plant-based - Tracking fat g - Reducing sugars - Increasing fiber	Diabetes Prevention Program [80]; 2010 US Dietary Guidelines [81]; American Cancer Society guidelines on nutrition and physical activity for cancer survivors [13]	Print material (American Institute for Cancer Research Nutrition and Physical Activity brochure) + 2 weight management sessions at Yale Centre for Cancer Survivor-ship Clinic	- Body weight ^a^ (kg): Int 1***; Int 2** - WC (cm): Int 1**; Int 2** - HC (cm): Int 1**; Int 2** - % body fat: Int 1*; Int 2 NS - LBM (kg): Int 1 NS; Int 2 NS - BMD (g/cm^2^): Int 1: NS; Int 2 NS - Weight loss (%): Int 1 CS; Int 2 CS	Combined intervention groups (Int 1 + Int 2): - Insulin (µU/mL): NS - Glucose (mg/dL): NS - CRP (mg/L): * - Leptin (ng/mL): NS - Adiponectin(µg/mg): NS - Il-6 (pg/mL): NS - TNF- α(pg/mg): NS	- % E from fat: Int 1: *; Int 2: * - Fiber (g/1000 kcal) Int 1*; Int 2* - F&V serves/day: Int 1*; Int 2* - Added sugar (g/1,000 kcal): Int 1 NS; Int 2 NS	NM	Int 1: **Y**: Weight loss Int 2: **Y**: Weight loss
Jen et al. (2004) [49] Djuric et al (2002) [50]	12 months; 12 months	Social Cognitive theory [82]; Individualized telephone counselling 0-3 M: Weekly × 12 3–6: Fortnightly × 6 6–12: Monthly × 6 24 sessions in total (time not reported) Plus, monthly weight loss information packet mailed NR	Weight loss by: - Decrease EI by 500–1000 kcal/d - Fat: 20–25% total E - Protein: 20% - F&V: ≥ 5 serves - Increase fiber through wholegrain	American Dietetic Association Exchange List for Meal Planning [83]	Print material (pamphlets)	- Body Weight (kg) - BMI (kg/m^2^) - Body Fat (%) - Weight loss (%) CS	- Total Chol ^a^ (mg/dL): * - LDL-C ^a^: NS - HDL-C ^a^: NS - Triglycerides ^a^: NS - Glucose ^a^ (mg/dL): NS - Insulin ^a^ (µU/mL): NS - HOMA^a^: NS - Leptin ^a^ (ng/mL): NS	- EI (kcal): NS - Fat Intake (%): NS	NM	**Y**: Total Chol **N**: Other lipids **N**: Insulin sensitivity **N**: Leptin
Reeves et al. (2017) [51]	6 months; 6 months	Social Cognitive theory [82]; Individualized telephone delivered counselling Portion control Reduced E density kJ counting 0-6W: Weekly × 6 6–18: Fortnightly 15–16 sessions in total NR	Weight loss by: - E deficit of 2000 kJ/day - Total fat < 30% - SFA < 7% - V: 5 serves/d - F: 2 serves/d	World Cancer Research Fund, American Institute for Cancer Research. Food, nutrition, physical activity and the prevention of cancer: a global perspective [84]	Print material (mailed feedback following baseline and 6-month assessment)	- Body weight ^a^ (kg): * - FFM (kg): NS - FM (kg): * - Fat (% body weight): NS - WC (cm): ** - HC (cm): *** - Weight loss (%) CS	NM	NM	- SF-36 PCS: NS - SF-36 MCS: NS - FACIT-F: NS - BIRS: NS	**Y:** Weight loss
Reeves et al. (2021) [55] Reeves et al (2016) [56]	12 months; 12 months	Social Cognitive theory [82]; Remote telephone coaching Healthy eating weight management Encouraging self-monitoring, goal setting Text messaging 0-6 m: 16 × calls 6-12 m: calls + text NR	Weight loss by reducing: - EI by 2000 kJ/d (to 5000–7500 kJ) - Portion size - E density - ≤ 30% E as fat - SFA < 7% - 10 g EtOH/d - 2 AFD serves/week - V: 5 serves/day - F: 2 serves/day	Guideline for the management of overweight and obesity in adults [85] Nutrition and physical activity guidelines for cancer survivors [13] WCRF guidelines for cancer prevention [17, 86]	Standard medical care Print materials (assessments and newsletter)	- Body weight ^a^ (kg): *** - Total fat mass (kg): *** - Total lean mass (kg): NS - WC (cm): ** - Weight loss (%): CS	- Fasting Plasma Glucose (mmol/L): * - Metabolic Syndrome risk score: ** - Triglycerides (mmol/L): NS - HDL-C (mmol/L): NS	NM	- Systolic BP: NS - Diastolic BP: NS - Musculoskeletal pain: ** - Body Image: * - Fatigue: NS - Menopausal symptoms: NS - Fear of cancer recurrence: NS - QoL Physical: ** - QoL Mental: NS	**Y**: Weight loss
Shaw et al. (2007a) [61]	24 weeks; 24 weeks	NR; Individualized dietary advice based on 7-day food diary No. of dietitian consultations not clear NR	Int 1: Weight loss - EI: 1-2 MJ/day - Reduce high fat/ refined CHO foods - Exchange lists Int 2: Low fat - at 20% of EI - List of 5 g fat exchange foods - Increase CHO to maintain EI	Clinical Practice Guidelines for the Care and Treatment of Breast Cancer: 11 Lymphedema [87]	Continue habitual diet	- Body weight ^a^ (kg) Int 1: ***; Int 2: NS - BMI Int 1: **; Int 2: NS - Skinfold thickness Int 1: NS; Int 2 NS - Weight loss (%) Int 1: CS; Int 2: NCS	NM	- EI (MJ) Int 1***; Int 2** - Fat (g) Int 1***; Int 2*** - Protein (g) Int 1**; Int 2 NS - CHO (g) Int 1 NS; Int 2 NS - % E from Fat Int 1**; Int 2***	- Excess arm volume ^a^ (%) Int 1: NS; Int 2: NS	**Int 1:** **N:** Excess arm volume **Y**: Weight loss **Int 2:** **N:** Excess arm volume **N:** Weight loss
Shaw et al. (2007b) [62]	12 weeks; 12 weeks	NR Individualized dietary advice based on participant usual meal pattern Food exchange lists to encourage variety No. of dietitian consultations not clear NR	- Weight loss through E deficit of 1000 kcal/day - Intake 1000–1200 kcal/day - Reduce foods containing fats and CHO	Clinical Practice Guidelines for the Care and Treatment of Breast Cancer: 11 Lymphedema [87]	Print material (Royal Marsden NHS Trust Patient Information Series Booklet No. 31 on healthy eating)	- Body weight (kg): * - BMI (kg/m2): * - Skinfold thickness (mm): NS - Weight loss (%): NCS	NM	- E (kcal/d): ** - Fat (g/d): ** - Protein (g/d): NS - CHO (g/d): ***	- Reduction in excess arm volume ^a^ (mL): **	**Y:** Reduction in excess arm volume **Y**: Weight loss
Prostate Cancer										
Baguley et al. (2021) [63] Baguley et al (2017) [64]	12 weeks; 12 weeks	Motivational Interviewing [88] as part of dietetic practice; Face-to-face Individualized MED-diet meeting estimated nutrient requirements and dietary preferences of each participant E requirement calculated using Harris-Benedict equation Fortnightly: 6 × 30–45 min over 12 weeks = 3 – 4.5 h	MED-diet: - SFA: < 10% E - F: 2 serves/day - V: 5 serves/day - Fiber: 30 g/day - Reduce/ eliminate red/ processed meats - Fish: 3 serves/week - Dairy: 2 serves/day - Nuts/seeds: 1serve/day - EtOH: ≤ 20 g/week - Energy %: CHO: 45–65; Fat:20–35; Prot: 15–25 - SFA: < 10% E BMI > 25 kg/m^2^: -Decrease EI by 2000–4000 kJ	Dietitians Association of Australia Best practice guidelines for the treatment of overweight and obesity in adults[89]	Usual medical care	- Total body mass (kg)*** - Lean mass (kg) NS - Fat mass (kg) NS - Weight loss (%): NCS	- Inflammatory markers: - IL-6 (ng/ml): NS - IL-8 (ng/ml): NS	- EI (MJ/d): NS - Protein (g/day): NS - Fat (g/day): * - SFA (g/day): *** - F (serves/day): * - V (serves/day): ***	- FACIT-F ^a^: ** - FACIT-G ^a^: ** - SF-36-VT ^a^: ** - SF-36-MCS ^a^: * - SF-36-PCS ^a^: NS - SF-36 subscales (Role function, Bodily pain, General, Social function, Role emotion): NS	**Y:** CRF **Y:** QoL by FACIT-G (but not SF-36) **Y:** weight loss
Mohamad et al. (2019) [65]	12 weeks; 12 weeks	NR Individually tailored dietary advice based on 24 h recall Initial 1 h group session 3 × phone calls over 12 weeks Supported to set personal goals Access to web-based resources NR	Reduce EI by - decreasing portion sizes, - Reduce high-E, high-fat, high-sugar foods - Reduce alcohol - Increase F&V and wholegrain	NR	No intervention (waitlist)	- Weight change ^a^ (kg): ** - Weight loss (%): NCS	NM	NM	- Global QoL score: ** - Functional QoL score: * - Symptoms QoL score: NS	**Y:** Weight loss
Colorectal Cancer										
Alavi et al. (2023) [45] Henriksen et al. (2017) [68]	6 months; 6 months	Motivational Interviewing [88]; Personalized face to face and phone counselling for nutrition-related cancer symptoms, weight and malnutrition Discount card for healthy foods; access to web page and email access; invited to cooking course and ‘inspiration day’ 2 × face to face + 2 phone consults NR	Norwegian dietary guidelines - Berries, F&V ≥ 500 g/day - Wholegrains 70-90 g/day - Fish 300 450 g/day - Red & processed meat ≤ 500 g/week - added sugar < 10 E% - Salt < 6 g/day - Attain/maintain BMI within normal range	Norwegian Dietary Guidelines [90]	Print material (booklet)	- Weight ^a^ (kg): * - FFM ^a^ (kg): NS - FM ^a^ (kg): * - FM ^a^ (%): * - FM/FFM ^a^: * - VAT: * - SAT: NS -Weight loss (%): NCS	NM	NM	NM	**Y:** Decreased weight gain **Y:** FM/FFM
	12 months; 12 months	As above plus: 3 × face to face + 3 phone consults NR				- Weight^a^ (kg): NS - FFM^a^ (kg): NS - Fat mass^a^ (kg): NS - FM^a^ (%):* - FM/FFM ^a^: * - VAT: NS - SAT: NS -Weight loss (%): NCS				**N:** Weight **Y**: FM/FFM
Ho et al. (2020) [69] Ho et al. (2013) [70]	12 months; 12 months	Theory of Planned Behavior [79]; The Health Action Process Approach [91]; Individual face to face consults: 2 over 4 months Motivational telephone calls every 2 weeks; Information pamphlets; quarterly group meetings NR	- Red/processed meat < 5 serves/week - Processed meat < 2 serves/week - Refined grains/d < 2 serves/day	NR	Print material (brochures)	NR	- Hemoglobin: NS	- Reduction in red/ processed meat: *** - Reduction in refined grains: ***	General QoL^a^: - SF-6D: NS - SF 12 PCS: NS - SF 12 MCS: NS CRC QoL^a^: - FACT-C: NS - FACT-G: NS - Anxiety^a^: NS - Depression^a^: **	**N:** General QoL **N:** CRC QoL **N:** Anxiety **Y:** Depression
Wang et al. (2022) [78]	6 months; 6 months	NR Customized nutritional intervention Home visit WeChat/telephone nutrition education 0&6 Months: Home visit Monthly: Video call through WeChat Over 6 months NR	-Prevent malnutrition/Adequate nutritional status -increase intake of nutrient dense foods - limit consumption of added sugars	Purpose developed dietary guide for CRC patients based on energy and nutrient requirements specified by National Health Commission of China	Standard care (no nutrition care)	- Weight change (kg): *** - BMI: NS - Calf circumference change: ***	NM	- EI (kcal/d): *** - Protein (g/day): *** - CHO (g/day): NS - Fat (g/day): NS	- PG-SGA^a^: ** - QoL Global: *** QoL Subscales: - Physical: ** - Role: *** - Social: *** - Emotional: * - Cognitive: NS Symptom scales: - Fatigue: *** - Pain: * - Nausea/ vomiting: NS - Dyspnea: ** - Insomnia: * - Appetite loss: * - Constipation: NS - Diarrhea: NS - FD: *	**Y:** PG-SGA **Y:** Weight gain

* *p* < 0.05; ** *p* < 0.01; *** p < 0.001

^a^ Primary outcome measure

^b^ Did not provide data necessary to calculate *p* values

Abbreviations: AFD = Alcohol free days; BIRS = Body image and relationships scale; BMD = Bone mineral density; BMI = Body mass index; BP = Blood pressure; C = Control; CHO = Carbohydrate; Chol = Cholesterol; CS = Clinically significant; CRC = Colorectal cancer; CRP = C-reactive protein; E = Energy; EI = Energy intake; EtOH = Alcohol; F = Fruit; FACIT-F = Functional assessment of chronic illness therapy – fatigue; FACIT-G = Functional assessment of chronic illness therapy – general; FACT-B = Functional assessment of cancer therapy-breast cancer; FACT-C = Functional assessment of cancer therapy-colorectal scale; FACT-G = Functional assessment of cancer therapy – general; FD = Financial difficulties; FFM Fat free mass; FM = Fat mass; HC = Hip circumference; HDL-C = High density lipoprotein cholesterol; HOMA = Homeostatic model assessment; IL-6 = Interleukin-6; IL-8 = Interleuken-8; I = Intervention; Int = Intervention; LDL-C = Low density lipoprotein cholesterol; LMB = Lean body mass; MED-diet = Mediterranean-style eating pattern; min = minutes; NCS = Non clinically significant; NM = Not measured; NR = Not reported; NS = Non-significant; PG-SGA = Patient generated – subjective global assessment; QoL = Quality of life; SAT = Subcutaneous adipose tissue; SF-12 MCS = 12-item short form health survey – mental component score; SF-12 PCS = 12-Item short form health survey – physical component score; SF-36 = 36-item short form health survey; SF-36 MCS = 36-item short-form health survey – mental component score; SF-36 PCS = 36-item short-form health survey – physical component score; SF-36 VT = 36-item short-form health survey – vitality; SF-6D = Six-dimensional health state short form; SFA = Saturated fat; TFN = Tumor necrosis factor; V = Vegetables; VAT = Visceral adipose tissue; WC = Waist circumference; WCRF = World cancer research fund

### Characteristics of included studies

The 12 included RCTs involved 1138 participants, (519 breast cancer; 75 prostate cancer; 544 colorectal cancer) ranging in age from 18 to 81 years. Seven studies provided interventions to breast cancer survivors [46, 48, 49, 51, 55, 61, 62], two studies to prostate cancer survivors [63, 65] and three to colorectal cancer survivors [45, 69, 78]. Studies were conducted in Australia (*n* = 3) [51, 55, 63], the United Kingdom (*n* = 3) [61, 62, 65], United States of America (*n* = 2) [48, 49], China (*n* = 2) [69, 78], Korea (*n* = 1) [46] and Norway (*n* = 1) [45]. The gender of participants varied according to cancer site with women recruited to the seven breast cancer studies [46, 48, 49, 51, 55, 61, 62]; men recruited to the two prostate cancer studies [63, 65] and the three colorectal cancer studies recruiting both men and women [45, 69, 78]. In the study by Cho and colleagues [46], *p* values could not be calculated using the published data provided. The two corresponding authors were contacted by email requesting additional data, however no response was received.

### Intervention description

Intervention durations varied from the shortest time of two months (*n* = 1) [46] to a maximum of 12 months (*n* = 4) [45, 49, 55, 69]. Alavi and colleagues [45] included a data end point at six months, then continued the intervention and had a second data endpoint at 12 months. All but two studies [45, 46, 48, 49, 51, 55, 63, 65, 69, 78] reported the number of dietitian sessions, which ranged from two sessions [46] to 24 sessions [49] (mean = 9.3), however only three studies also reported session duration allowing the calculation of total dietitian time per participant. The intervention with breast cancer survivors by Cho and colleagues [46] comprised 1.3 h (40 min per session) plus a 60-min cooking class across two months, Baguley and colleagues [63] delivered 3–4.5 h (45 min per session) of dietitian time in their three month intervention with prostate cancer survivors, while Harrigan and colleagues [48] delivered 5.5 h (30 min per session) of dietitian time over six months to breast cancer survivors. While all interventions provided individualized counselling with a dietitian, the mode of delivery varied. Four studies including one arm of the Harrigan study used face to face counselling [45, 48, 63, 69]. Three studies [45, 65, 69] implemented group sessions in addition to individual counselling. Four studies including one arm of the Harrigan study [48, 49, 51, 55], used only telephone calls to deliver the intervention; two studies [45, 69] delivered their intervention using a combination of face-to-face and telephone consultations. Wang and colleagues’ [78] intervention consisted of WeChat videos and two home visits by the dietitian as well as WeChat app/telephone for educational purposes. Two studies added cooking classes to the dietitian delivered intervention [45, 46]. Three studies did not clarify the delivery mode of individual counselling [46, 61, 62].

Comparator groups received print materials, standard or usual care, were waitlisted or received no intervention. The majority of studies (*n* = 8) provided print materials such as newsletters, brochures, dietary guidelines as well as general and healthy eating guides to their comparator groups [45, 46, 48, 49, 51, 55, 62, 69]. The four studies providing standard care described this as standard medical care [55, 63] a two-session weight management program [48] or a follow-up telephone call after six months [78].Two studies [48, 55] combined standard care with print materials. One study waitlisted participants who later received the intervention [65], while in the final study [61] control participants received no care or materials.

### Dietary goals

The goals of dietary intervention varied in alignment with the primary outcome measures. Of the seven breast cancer studies, all but one [46] had a dietary goal of weight loss as a primary outcome measure [48, 49, 51, 55, 61, 62]. The dietitians advised participants on how to decrease energy intake through decreasing dietary fat and sugar consumption [48, 49, 51, 55, 61, 62]. The dietary goal in the Cho study [46] which had a normal weight population was to increase fruit and vegetable intake. Of the two prostate cancer studies, one [63] used the Mediterranean diet to improve QoL and cancer-related fatigue (CRF), and the other study [65] had weight loss as a goal and the primary outcome measure of weight change. Of the three colorectal cancer studies, the one conducted in Norway with a mean sample BMI of 27 kg/m^2^ [45] aimed to improve body composition (proportion of fat mass to fat free mass) and provided weight reduction advice to those participants with a BMI at or above 25 kg/m^2^. The other two colorectal cancer studies [69, 78] were conducted in China in samples with a mean BMI in the healthy weight range, and therefore did not set weight loss goals. The dietary goal for those studies was to improve nutritional status to improve quality of life, with the study by Ho and colleagues [69] seeking to decrease western style dietary habits to achieve this.

### Guidelines

Nine of the twelve studies used guidelines as a basis for the dietary intervention [45, 48, 49, 51, 55, 61–63, 78]. Three studies used nutrition guidelines tailored explicitly to cancer survivors: the American Cancer Society guidelines on nutrition and physical activity for cancer survivors [48, 55] and a purpose developed dietary guide for colorectal patients based on energy and nutrient requirements specified by the National Health Commissioner of China [78]. Shaw and colleagues [61, 62] used the clinical guidelines for the care and treatment of breast cancer: lymphedema which encourage ideal body weight. Two studies used cancer prevention guidelines by the WCRF [51, 55]. Three studies used guidelines for overweight, obesity and weight loss management [49, 55, 63] Two studies [45, 48] used national dietary guidelines, Norwegian and USA respectively. Two studies [48, 55] used three different sets of guidelines as the basis for their intervention.

### Behavior change theories

Six studies reported the use behavior change theories to underpin their interventions [45, 48, 49, 51, 55, 69]. Three [49, 51, 55] used Social Cognitive Theory [82], one [48] used the Theory of Planned Behavior [79], one [45] used Motivational Interviewing [88] and one [69] used both the Theory of Planned Behavior and The Health Action Process Approach [91].

## Results of individual studies

### Anthropometry

Intentional weight loss was sought and achieved in 10 studies [45, 46, 48, 49, 51, 55, 61–63, 65]. Of the seven studies focused on breast cancer survivors, five measured weight loss as a primary outcome [45, 48, 51, 55, 65] with Jen and colleagues measuring weight loss as a secondary outcome to metabolic measures, and four reported percentage body fat [48, 49, 51, 55]. Only two of the breast cancer studies [48, 55] reporting weight loss also reported percentage weight loss. Calculations performed with the remaining four [49, 51, 61, 62], of within-group percentage weight loss, resulted in five studies [48, 49, 51, 55, 61] showing clinically significant mean weight loss of between 5%—10% of baseline weight. Six breast cancer studies [48, 49, 51, 55, 61, 62] were subjected to meta-analyses (reported below). Both prostate cancer interventions [63, 65] had weight reduction goals and listed weight as a primary outcome measure. Both studies reported significant weight loss over control. Of the three studies with colorectal cancer survivors [45, 69, 78] only the study by Alavi and colleagues [45] had a weight-related dietary goal, which was to keep BMI within a healthy range. While both groups gained weight, the intervention group gained less weight and less fat mass by six months than the control, although the difference between the two groups was only significant for percentage of body fat after the full 12 month intervention [45].

### Quality of life

Two studies [63, 69] cited quality of life as their primary outcome measure, and another five included QoL measures [46, 51, 55, 65, 78]. Three studies [46, 65, 69] used cancer site specific tools to assess QoL, with the remainder using generic cancer QoL measures [55, 65, 69, 78]. Of these, three studies [51, 55, 63] used a multidimensional health measure for chronic diseases, four studies [51, 55, 63, 69] used general health QoL tools, two studies [51, 55] the body image and relationship scale (BIRS) and just one study [69] used the hospital anxiety and depression scale (HADS). Five studies [51, 55, 63, 65, 69] used two or more tools. Only three of the seven RCTs with breast cancer survivors measured QoL [46, 51, 55] and only Reeves and colleagues [55] found that QoL as measured by the patient-reported outcome measurement information system (PROMIS) and BIRS improved with their intervention outcomes. Both prostate cancer studies reported QoL with Baguley and colleagues [63] finding significant intervention effects shown as measured with the functional assessment of chronic illness therapy – fatigue (FACIT-F), the functional assessment of chronic illness therapy – general (FACIT-G), the 36-item short-form health survey – vitality (SF-36-VT), and the 36-item short-form health survey – mental component score (SF-36-MCS). Mohamad and colleagues [65] found significant differences in some aspects of QoL compared with control when measured by European organization for research and treatment of cancer for assessing the generic aspects of QoL (EORTCQLQ-C30) and the European organization for research and treatment of cancer specific for prostate cancer (EORTCQLQ-PR25). Two of the three colorectal cancer studies also reported improved QoL in their intervention group over control, as measured in the study by Wang and colleagues [78] with the EORTCQLQ-C30. Ho and colleagues [69] found significant intervention effects in levels of depression as measured by HADS.

### Dietary intake

Eight studies reported measuring dietary intake [46, 48, 49, 61–63, 69, 78]. Cho and colleagues [46] used results from a 3-day food diary to assess fruit and vegetable intake as their primary outcome measure. They reported a significantly greater increase in fruit and vegetable intake in their intervention group over control although this statistical difference could not be verified by the data provided. Other studies had dietary intake as secondary outcomes and collected measures using food frequency questionnaires [48, 69], 3 and 7- day food records [46, 49, 61, 62], 24 h recall [78] and 1 month diet history [63]. They showed significant intervention effects for fruit and vegetable intake [48, 63], fiber intake [48, 63], fat intake [48, 61–63], caloric intake [61, 62], carbohydrate intake [62] and saturated fat intake [63]. Wang and colleagues [78] showed a significant increase in caloric intake compared to control in their intervention aimed at preventing malnutrition in colorectal cancer survivors.

### Biochemistry

Of the six studies [46, 48, 49, 55, 63, 69], conducting blood analysis only Jen and colleagues [49] measured the biochemical outcomes associated with weight loss as a primary outcome. They found a significant reduction in total cholesterol levels above control in their intervention with breast cancer survivors [49]. Similarly, Reeves and colleagues [55] found a significant improvement in fasting plasma glucose levels and metabolic syndrome risk score above control in the breast cancer survivors in their weight loss intervention. Cho and colleagues [46] reported significant intervention effects for serum carotene and vitamin A in favor of the intervention. Two studies [48, 63] reported on serum inflammatory markers, with Harrigan and colleagues [48] finding a significant decrease in C-reactive protein when compared with control, however neither study found a significant decrease in interleukin 6 and interleukin 8.

### Clinical

Reeves and colleagues [55] found significant improvement in arthralgia, however found non-significant intervention outcomes in both systolic and diastolic blood pressure and menopausal symptoms as measured by Greene climacteric scale [57]. In their first study, comparing the effectiveness of two different diets (weight reduction and low fat) against a control group, Shaw and colleagues [61] found a reduction in breast cancer-related lymphedema in participants achieving weight loss. Their subsequent study [62] focused on the dietary goal of weight reduction, and found a significant decrease in lymphedematous excess arm volume due to the intervention.

## Results of meta-analyses

**Fig. 2:**
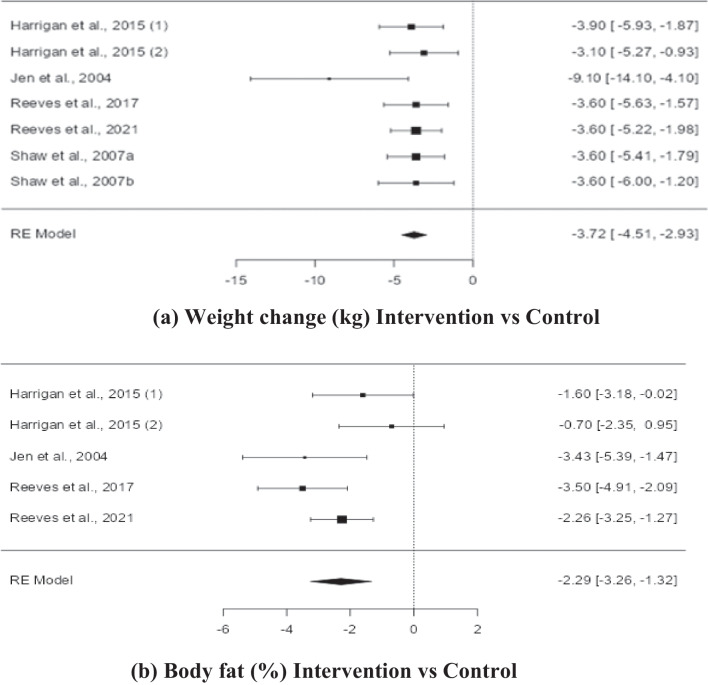
Forest plots depicting comparisons for dietitian counselling vs control for change in (a) weight (kg) and (b) body fat (%)

Meta-analyses were able to be conducted on six studies of female breast cancer survivors (n = 458), representing seven interventions with weight reduction as a dietary goal and measures of body weight change [48, 49, 51, 55, 61, 62]. The mean baseline BMI for these samples were in the overweight [48, 51, 55, 61, 62] or obese [49] range. The results are shown in Fig. 2a. Four of these studies [48, 49, 51, 55] also measured change in body fat percentage, and a second meta-analysis was conducted on this measure with 370 participants (see Fig. 2b).

The observed mean differences in weight change ranged from -9.1000 to -3.1000 and observed mean differences in percentage body fat ranged from -3.5000 to -0.7000 with all the estimates favoring intervention. The estimated average mean difference based on the random-effects model for weight was -3.7172 (95% CI: -4.5084 to -2.9259), and for percentage body fat was 2.2895 (95% CI: -3.2561 to -1.3229) therefore, the average outcome differed significantly from zero (z = -9.2068, *p* < 0.0001) for weight loss and (z = -4.8425, *p* < 0.001) for percentage body fat (Fig. 2). According to the Q-test, there was no significant amount of heterogeneity in the true outcomes for weight (Q = 4.8561, *p* = 0.5624, tau^2^ = 0.0000, I^2^ = 0.0000%) however some heterogeneity may still be present in the true outcomes for percentage body fat (Q = 8.4216, *p* = 0.0773, tau^2^ = 0.000, I^2^ = 53.8710%). A 95% prediction interval for the true outcomes is given by -4.5085 to -2.9258 for weight and -4.1304 to -0.4486 for percentage body fat, so while there may be some heterogeneity, the true outcomes of the studies are generally in the same direction as the estimated average outcome for both meta-analyses. An examination of the studentized residuals revealed that none of the studies had a value larger than ± 2.6901 for weight and ± 2.5758 for percentage body fat, hence there was no indication of outliers in the context of this model. According to the Cook's distances, none of the studies could be considered to be overly influential. Neither the rank correlation nor the regression test indicated any funnel plot asymmetry (*p* = 0.2389 and *p* = 0.0646, respectively) for weight and (*p* = 0.8167 and *p* = 0.9630, respectively) for percentage body fat.

## Risk of bias

**Fig. 3:**
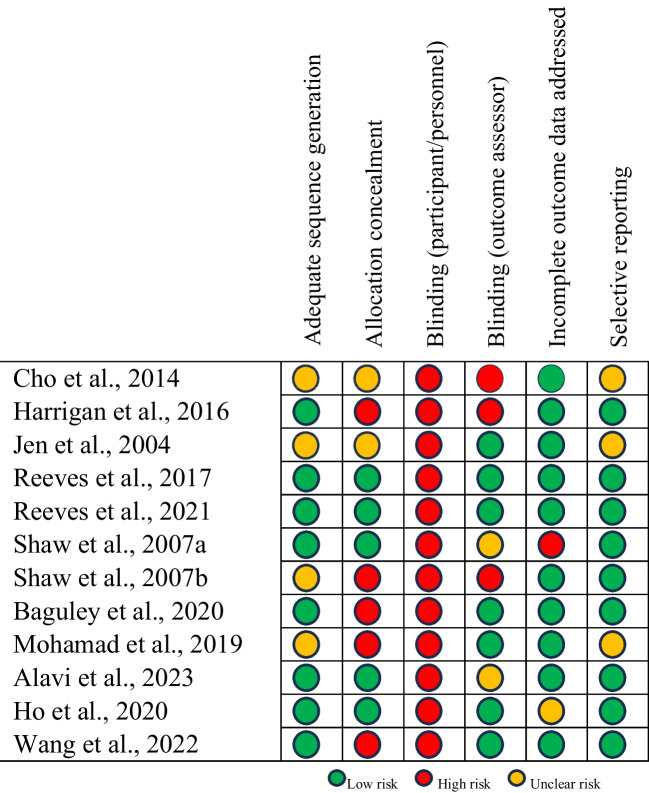
Risk of bias summary diagram

A summary of the risk of bias assessment is displayed in Fig. 3. All included RCTs displayed some areas of high or unclear risk of bias. Adequate sequence generation revealed eight studies [45, 48, 51, 55, 61, 63, 69, 78] with low risk and four [46, 49, 62, 65] with unclear risk, not adequately mentioning the randomization process. Allocation concealment was not well described in eight studies, with five studies [48, 62, 63, 65, 78] showing high risk, two [46, 49] showing unclear risk and five [45, 51, 55, 61, 69] showing low risk. Given the nature of dietetic interventions, double blinding is challenging, resulting in all studies in this domain being at high risk of bias. Blinding of outcome assessors was described in seven studies [49, 51, 55, 63, 65, 69, 78] and considered low risk. Three studies [46, 48, 62] were considered high risk and two [45, 61] were at unclear risk of bias due to ambiguity surrounding blinding of outcome assessment. All but two studies received a low-risk rating for incomplete addressing of outcome data, with one study [69] at unclear risk and the other [61] at high risk. Selective reporting resulted in nine studies [45, 48, 51, 55, 61–63, 69, 78] being considered low risk and the remaining three [46, 49, 65] being at unclear risk.

## Discussion

This systematic review and meta-analysis add to the current evidence base by specifically focusing on the outcomes of dietetic interventions with adult survivors of cancer occurring in the breast, prostate, and bowel. While there is existing literature on the importance of care provided to cancer patients in the acute care setting [92, 93], this study was the first to synthesize the evidence evaluating nutritional care provided exclusively by dietitians to adult cancer survivors post active treatment. While dietitian interventions were only reported for these three cancer types, the study participants spanned the diversity of nutritional statuses found in cancer, from undernutrition to obesity. Most of the interventions showed a positive effect for the weight related indices, on dietary intake, blood parameters, and some for clinical measures and quality of life.

A healthy weight supports overall health and minimizes the risk of further chronic disease making weight management a key component of comprehensive survivorship care [94]. The nutrition and physical activity guidelines for cancer survivors [13] state that any weight loss is beneficial with significant health benefits achievable with weight loss of between 5 to 10%. However, there is some evidence from the Women’s Intervention Nutrition Study that a low-fat diet resulting in a 2.7 kg weight loss (4% initial body weight) reduced the risk of recurrence among postmenopausal breast cancer survivors [95]. Dietetic effectiveness was consistently demonstrated in the interventions where weight loss was the primary outcome. Even more importantly, this study quantifies the effect of dietitian intervention in breast cancer survivors. The meta-analyses demonstrated that dietetic interventions of 12 months or less were able to achieve an intentional weight loss of 3.7 kg (-4.51, -2.93) and body fat decrease of 2.3% (-3.26, -1.32) when compared with control. This reflects results of a systematic literature review and meta-analysis conducted by Williams and colleagues [96] exploring the effectiveness of dietitians in weight management in adults where 1.03 kg weight loss was achieved above usual care. Similarly, results of a systematic review and meta-analysis by Sun and colleagues on weight loss interventions delivered by both dietitians and non-dietitians, found participants in dietitian delivered interventions lost a mean 2.1 kg, resulting in 1 kg greater weight loss than those in non-dietitian delivered interventions [97]. Hawkins and colleagues [98] in a cross-sectional survey of 7,903 participants suggest that cancer survivors may be more motivated to make positive behavior changes, which may account for the breast cancer survivors achieving more than three times the weight loss of the patients with chronic disease in the earlier reviews.

Optimizing body composition in overweight cancer survivors by reducing the proportion of body fat can reduce cancer-related symptoms [99] and improve prognosis [100]. The meta-analysis for body fat percentage suggests that the weight loss observed in these overweight and obese cancer survivors reflects a desirable change in body composition, rather than a predominant loss of lean body mass. This is important given that sarcopenia – a loss of muscle mass and function [101] – is seen in cancer survivors of all sizes [102] often caused by the direct effect of chemotherapy on muscle, lack of exercise and impaired nutrition [103]. Low muscle mass can be improved through nutritional strategies such as higher level of protein intake [104] which could benefit progress-free survival [105]. The dietary advice in the interventions included in the meta-analyses focused on decreasing energy intake from fats and sugars, and only one [49] sought to increase protein to at least 20% of energy. There may be scope for interventions to increase focus on protein intake to promote favorable body composition in all cancer survivors, not just those who are undernourished.

Interventions should be based on the best available evidence. Evidence-based guidelines state that cancer survivors need to maintain a healthy weight, integral to a healthy diet and lifestyle [13, 18]. The American Cancer Society nutrition and physical activity guideline for cancer survivors provides cancer specific guidelines for several cancer types [13]. While seven of the included RCTs designed their interventions using dietary guidelines, only three utilized guidelines specific to cancer survivors [48, 55, 78]. Lack of consistency in guideline use in the included studies may indicate the need for guidelines to be better tailored to the primary care setting and the needs of post-treatment cancer survivors. Primary care dietitians need to be able to provide consistent and evidence-based dietary advice founded on relevant cancer guidelines. Survivorship care post-treatment can be complex due to the need to address the type of cancer, the aftereffects of treatment, the potential for cancer recurrence, the increased risk of chronic disease and to promote QoL. Research into the use of guidelines in primary care dietetics could provide valuable insights in determining the current usage of guidelines and assess the adequacy of existing guidelines in addressing the nutritional care of cancer survivors.

Dietitians use their expert nutrition knowledge to improve health outcomes by achieving changes in dietary behavior [106]. A systematic review conducted by Rigby and colleagues found that nutrition interventions underpinned by behavior change theories to be more effective in improving health outcomes than those that are not [107]. Behavior change theory combined with behavior change techniques added to diet counselling have been shown to have positive effects on cancer survivors [108]. Despite this evidence, only half of the included studies reported basing their intervention on behavior change theory. In an examination of methodological quality of 27 RCTs reporting dietetic intervention, Ball and colleagues [109] categorized counselling in behavior change as influencing long-term success and argued for increased rigor in reporting dietetic interventions.

Dietitians achieve behavior change through a diverse range of strategies, tailoring dietary intake to patient needs. Dietitians in the included interventions engaged cancer survivors in individual, group or telephone counselling sessions, social media, and cooking classes. Given the heterogeneity in counselling techniques found in this review, it was not possible to ascertain whether one was more effective than the other. One study [48] compared two intervention arms, face-to-face and telephone consultation and found no significant difference in the primary outcome of weight loss. Only one study in this review [78] used video calls (combined with telephone calls) for one-on-one counseling and showed improvement in post treatment health status. With more dietitians, particularly in primary care, utilizing online technology [110], there is scope to investigate the impact of video calls in providing care to cancer survivors. The number of dietitian visits ranged from two to 24, with no apparent difference in results. Unfortunately, only three of the included studies reported the amount of time participants spent with dietitians, allowing the calculation of total time spent. A lack of detailed reporting of time spent with the dietitian was also found in the review by Mitchell and colleagues [20]. Future studies should record and report this simple measure to allow cost-effectiveness to be assessed. Intervention duration ranged from two to 12 months. Further research is needed to ascertain whether gains made during interventions are maintained beyond a 12-month period.

A variety of outcomes were measured in the included studies. Associations between weight loss and improvements in metabolic diseases are well established [111] and are of particular importance to cancer survivors who face increased risk of metabolic disorders. Studies in this review examined various blood measures including cholesterol, serum inflammatory markers, carotene, vitamin A and metabolic disease. Metabolic syndrome is characterized by elevated levels in three of five variables: triglycerides, cholesterol, fasting glucose blood pressure and waist circumference, and has been correlated with adverse outcomes in women with early breast cancer [112]. A systematic review and meta-analysis by Ross and colleagues [113] found that dietetic consultations in primary care were effective at lowering triglyceride levels and at least as effective as control for improving cholesterol levels in high risk individuals. This highlights the need for healthcare practitioners to consider blood analysis and referrals to dietetic care for cancer survivors who have metabolic disorders with or without weight related issues.

While not all patients are cured of cancer, many live with the disease over time and experience short- or long-term effects [114]. Cancer related fatigue (CRF) is the most prevalent after effect of cancer and impacts greatly on QoL [115]. CRF was assessed in three of the interventions in this review. Tools used in cancer studies often tend to measure health related QoL and Health Status rather than QoL per se [116]. To capture the complexity of elements and subjectivity of QoL, multiple measures and tools may be required, aimed at general health or be disease specific [117]. The studies included in this review measured QoL through a variety of measures, encompassing physical and mental well-being. The most widely used tools to assess QoL in cancer survivors are the FACT-G, and the EORTC QLQ-C30 [118]. FACT-G was used in two included studies [63, 69] and EORTC QLQ-C30 in a further two [65, 78]. Identifying commonly experienced aftereffects such as CRF can lead to benefits achievable through nutritional interventions.

There remains a need for more well-designed randomized control trials covering different cancer types other than breast, prostate, and bowel, as well as additional trials with these cancers to provide more data to meta-analyze. There is some published evidence regarding the cost effectiveness of dietitians. Sun and colleagues [97] found that the cost of interventions conducted by dietitians was lower than interventions delivered by non-dietitians with a mean cost per kg from $34 over six months to $1005.36 over 12 months. Additionally, Howatson and colleagues [119] found a saving of between NZ$ 5.50—$99 for the New Zealand healthcare system. This monetary value was calculated not only on the lower cost of dietetic consultations but also included the impact of a reduced number of yearly medical visits by people who see a dietitian. However, cost analysis can be challenging due to studies rarely including cost data, with none of the studies in our meta-analysis having done so. As emphasized in studies by Sun [97] and Williams [96] there is a need for more interventions conducting cost effectiveness analysis, clearly documenting dietitian time and associated costs.

Only two [48, 55] of the RCTs demonstrating weight loss in our meta-analysis reported on effect maintenance. Harrigan and colleagues [48] assessed weight loss at 12 months (six months post intervention) by self-report. Weight loss from baseline in both intervention arms was deemed clinically significant at 6.3% and 7.7% respectively. Reeves and colleagues [55] followed participants to 18 months (six months post intervention) and found some weight regain but the loss from baseline remained statistically significant (*p* = 0.007) at 3.7% more than usual care. Studies following participants for at least 12 months and preferably 24 months post intervention will be important to determine whether intervention effects are maintained.

The strengths of this review include the use of a systematic process and rigorous protocol in line with PRISMA recommendations. The addition of meta-analyses allowed in-depth statistical analysis of weight loss and percentage body fat in breast cancer patients, with low heterogeneity of both meta-analyses giving confidence in the summary of results. Limitations included restricting search dates to 2004 and after, however this gave the opportunity to focus on studies influenced by seminal works and reports, with analysis of contemporary data. Language was restricted to English due to researchers’ language limitations. Some publication bias may have occurred due to the exclusive inclusion of RCTs. It is acknowledged that a larger body of work exists on nutrition interventions with cancer survivors that have shown similar effects on weight loss. However, these included interventions with physical activity, and nutrition components that were not standardized and/or delivered by people with varying levels of training, making it difficult to assess the precise contribution of expert dietary intervention. While we acknowledge that physical activity should be part of cancer care, this review focused specifically on interventions conducted by credentialled dietitians in order to reflect the role of primary care dietitians.

## Conclusion

This systematic review and meta-analyses demonstrated a positive effect of dietitian intervention on weight and body fat percentage in overweight and obese breast cancer survivors at levels likely to be clinically significant. There was not significant data to conduct meta-analysis for other outcome variables or for the other cancer types, prostate and colorectal, although most of the individual RCTs suggested a positive effect of dietitian intervention. These findings could be used to advocate for a role for primary care dietitians in providing long term management to cancer survivors, particularly for weight management in breast cancer survivors. There remains a need for well-designed RCTs that clearly report time spent in dietetic consultation, conducted with survivors of cancer types other than breast, prostate and bowel. Studies investigating access to dietetic care for cancer survivors and models of care in the post-active treatment stage of cancer survivorship will also be important to achieve better health outcomes for this growing population.

## Supplementary Information

Below is the link to the electronic supplementary material.

Supplementary file1 (DOCX 59 KB)

## Data Availability

The authors confirm that the data generated or analyzed in this study are available within the article and its supplementary materials.
