# Supplementary material for: Effectiveness of dietetic care for cancer survivors in the primary care setting: A systematic review and meta-analysis of randomized controlled trials
Source: J Cancer Surviv. 2024 May 6;19(5):1694–725. doi: 10.1007/s11764-024-01583-6 (PMC12460552; doi:10.1007/s11764-024-01583-6)
Supplement: Supplementary file 1 — Supplementary file1 (DOCX 59 KB) [file 11764_2024_1583_MOESM1_ESM.docx]

**Appendix 1**

Eligibility Criteria using the PICOS Framework

|  | Included | Excluded |
| --- | --- | --- |
| Population | Adult cancer survivors (aged ≥18 years) of all cancer types who have completed the active treatment phase of cancer treatment (surgery, radiology, chemotherapy). | Cancer survivors (aged <18 years) or adults still undergoing active treatment. |
| Intervention | Nutrition care for the management/treatment of cancer survivors exclusively by a dietitian in a primary care setting including community, and private practice. | Nutrition care provided by non-dietitians or provided by dietitians in a hospital setting or during active treatment including outpatient services, or where the dietetic intervention cannot be separated from other interventions. |
| Comparator | Usual care, minimal care, or no intervention control. | Where intervention and comparator both use same level of dietetic care. |
| Outcome | Anthropometric measures   - weight, height, BMI, waist circumference, waist-to-hip ratio, skinfold thickness.   Clinical indicators   - biomarkers: cholesterol, triglycerides, blood glucose levels, inflammation, carotenoids   Dietary intake   - dietary behaviors.   Quality of Life. |  |
| Study | Randomized controlled trial. | Non- Randomized controlled trial. |

Abbreviations: BMI = Body mass index

**Appendix 2**

| **Database Search Strategy** |
| --- |
| 1. ‘neoplasm’ or ‘cancer’ or ‘oncology’ or ‘survivor*’ or ‘cancer survivor*’ or ‘oncology survivor’ |
| 1. ‘dietitian’ or ‘dietician’ or ‘dietetics’ or ‘nutritionist’ or ‘diet advice’ or ‘nutrition advice’ |
| 1. ‘consult*’ or ‘referral’ or ‘private practice’ or ‘counsel*’ or ‘interview’ or ‘advice’ or ‘outpatient’ or ‘clinic’ or ‘primary care’ or ‘primary health care’ [MeSH heading], ‘community health’ |
| 1. 1 and 2 and 3 |
| Limit to English language and humans |
| Date restriction: 2004-2023 |

**Appendix 3**

**Meta-analysis for weight loss**

| Random-Effects Model (k = 7) | | | | | | | | | | | | | |
| --- | --- | --- | --- | --- | --- | --- | --- | --- | --- | --- | --- | --- | --- |
|  | | **Estimate** | | **se** | | **Z** | | **p** | | **CI Lower Bound** | | **CI Upper Bound** | |
| Intercept |  | -3.72 |  | 0.404 |  | -9.21 |  | < .001 |  | -4.508 |  | -2.926 |  |
|  |  | . |  | . |  | . |  | . |  | . |  | . |  |
| Note. Tau² Estimator: Restricted Maximum-Likelihood | | | | | | | | | | | | | |
|  | | | | | | | | | | | | | |

| Heterogeneity Statistics | | | | | | | | | | | | | | | |
| --- | --- | --- | --- | --- | --- | --- | --- | --- | --- | --- | --- | --- | --- | --- | --- |
| **Tau** | | **Tau²** | | **I²** | | **H²** | | **R²** | | **df** | | **Q** | | **p** | |
| 0.002 |  | 0 (SE= 0.625 ) |  | 0% |  | 1.000 |  | . |  | 6.000 |  | 4.856 |  | 0.562 |  |
|  | | | | | | | | | | | | | | | |

| Publication Bias Assessment | | | | | |
| --- | --- | --- | --- | --- | --- |
| **Test Name** | | **value** | | **p** | |
| Fail-Safe N |  | 221.000 |  | < .001 |  |
| Begg and Mazumdar Rank Correlation |  | -0.429 |  | 0.239 |  |
| Egger's Regression |  | -1.849 |  | 0.065 |  |
| Trim and Fill Number of Studies |  | 0.000 |  | . |  |
| Note. Fail-safe N Calculation Using the Rosenthal Approach | | | | | |
|  | | | | | |


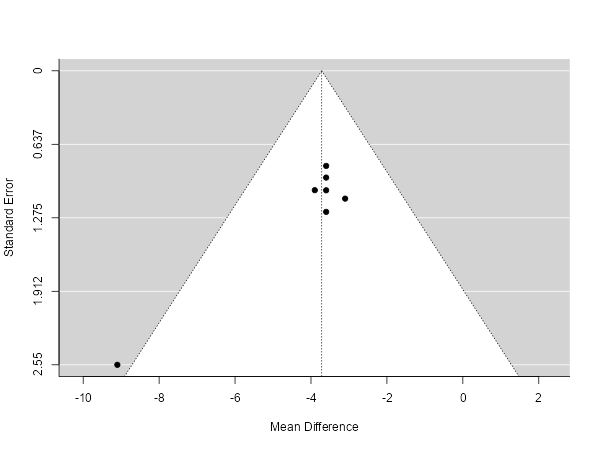


**Funnel plot** depicting heterogeneity of included studies for weight loss.

**Appendix 4**

**Meta-analysis for % Body Fat**

| Random-Effects Model (k = 5) | | | | | | | | | | | | | |
| --- | --- | --- | --- | --- | --- | --- | --- | --- | --- | --- | --- | --- | --- |
|  | | **Estimate** | | **se** | | **Z** | | **p** | | **CI Lower Bound** | | **CI Upper Bound** | |
| Intercept |  | -2.29 |  | 0.493 |  | -4.64 |  | < .001 |  | -3.256 |  | -1.323 |  |
|  |  | . |  | . |  | . |  | . |  | . |  | . |  |
| Note. Tau² Estimator: Restricted Maximum-Likelihood | | | | | | | | | | | | | |
|  | | | | | | | | | | | | | |

| Heterogeneity Statistics | | | | | | | | | | | | | | | |
| --- | --- | --- | --- | --- | --- | --- | --- | --- | --- | --- | --- | --- | --- | --- | --- |
| **Tau** | | **Tau²** | | **I²** | | **H²** | | **R²** | | **df** | | **Q** | | **p** | |
| 0.799 |  | 0.6389 (SE= 0.8564 ) |  | 53.87% |  | 2.168 |  | . |  | 4.000 |  | 8.422 |  | 0.077 |  |
|  | | | | | | | | | | | | | | | |

| Publication Bias Assessment | | | | | |
| --- | --- | --- | --- | --- | --- |
| **Test Name** | | **value** | | **p** | |
| Fail-Safe N |  | 85.000 |  | < .001 |  |
| Begg and Mazumdar Rank Correlation |  | 0.200 |  | 0.817 |  |
| Egger's Regression |  | -0.046 |  | 0.963 |  |
| Trim and Fill Number of Studies |  | 0.000 |  | . |  |
| Note. Fail-safe N Calculation Using the Rosenthal Approach | | | | | |
|  | | | | | |


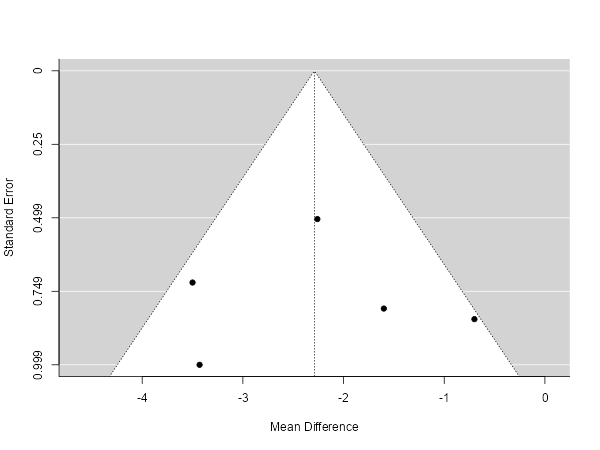


**Funnel plot** depicting heterogeneity of included studies for % Body Fat.
